# Farrerol Alleviates Myocardial Ischemia/Reperfusion Injury by Targeting Macrophages and NLRP3

**DOI:** 10.3389/fphar.2022.879232

**Published:** 2022-04-13

**Authors:** Lin Zhou, Shuhui Yang, Xiaoming Zou

**Affiliations:** ^1^ The Fifth Affiliated Hospital, Southern Medical University, Guangzhou, China; ^2^ Department of Thoracic Surgery, Yuebei People’s Hospital Affiliated to Shantou University Medical College, Shaoguan, China; ^3^ Department of Pathology, Yuebei People’s Hospital Affiliated to Shantou University Medical College, Shaoguan, China

**Keywords:** myocardial ischemia/reperfusion, farrerol, macrophage, NLRP3, NEK7

## Abstract

Myocardial ischemia/reperfusion (I/R) injury is associated with high mortality and morbidity, however, it has no curative treatment. Farrerol (FA), an active compound extracted from rhododendron, has antibacterial, anti-inflammatory, and antioxidant activities, but its effect and mechanism of FA in I/R injury remain unclear. Here, we found that FA alleviated myocardial I/R *in vivo*, and decreased the secretion of myocardial injury factors (CK-MB, LDH, troponin-1, and NT-proBNP) while inhibiting the release of inflammatory factors (IL-1β, IL-6, and TNF-α). FA could also alleviate excessive oxidative stress by elevating the level of antioxidant enzymes and reducing oxidation products; and decreased reduced the expression of apoptosis-associated proteins (cleaved caspase-3, Bax, and Bcl-2). However, inhibiting the autophagic pathway or knocking out the *Nrf2* gene did not eliminate the myocardial protective effect of FA, but interestingly, macrophage clearance and *Nlrp3* deficiency effectively blocked the myocardial protective effect of FA. In addition, FA suppressed NLRP3 inflammasome activation by interfering with NLRP3 and NEK7. In conclusion, these results support drug-targeted macrophage therapy for myocardial I/R and indicate that FA may be used as an immunomodulator in clinical therapy for myocardial I/R.

## Introduction

Myocardial ischemia/reperfusion (I/R) injury is a pathological process of progressive aggravation of tissue injury when the ischemic myocardium is restored to normal perfusion after a period of partial or complete acute coronary artery occlusion. According to the World Health Statistics 2021 report, 33.2 million deaths from the four major chronic diseases (cancer, cardiovascular disease (CVD), diabetes, and chronic respiratory disease) occurred in 2019; of which 17.9 million died from cardiovascular disease, ranking the highest mortality rate. The previous studies found that myocardial I/R injury is a major obstacle in CVD therapy (Maarman et al., 2012; Ait-Aissa et al., 2019). Currently, myocardial infarction is the leading cause of CVD-associated death worldwide (Zagidullin et al., 2020). Myocardial I/R causes oxidative stress and inflammation, thus increasing the infarct size and aggravating myocardial injury (Venkatachalam et al., 2009; Yeung et al., 2015). To date, clinical treatment methods to avoid myocardial I/R injury are lacking, thus hindering coronary heart disease therapy. Therefore, studying the pathophysiological mechanism of I/R is important to find effective prevention and treatment methods, and develop new therapeutic drugs to improve the prognosis of patients with coronary heart disease.

Many studies have examined the mechanism of I/R injury; which is mainly thought to be associated with the inflammatory effects of the massive production of intracellular oxygen free radicals, calcium overloading, the inflammatory effects of leukocytes, and the absence of high-energy phosphoric compounds (Kalogeris et al., 2012; Yan et al., 2020). I/R injury is characterized by the massive generation of free radicals, mitochondrial dysfunction, increased lipid peroxides, myocardial cell necrosis, apoptosis, weakened left ventricular contractility, malignant arrhythmias, decreased indoor pressure, and myocardial function inhibition (Ibáñez et al., 2015; Dong et al., 2019). Previous studies have shown that drug-activated nuclear factor erythroid 2-associated factor 2 (NRF2) protects against myocardial oxidative stress and myocardial injury (Shen et al., 2019). Autophagy protects I/R-induced myocardial injury by activating the AMPK protein kinase, thus decreasing apoptosis (Shi et al., 2019). In addition, blocking the release of proinflammatory cytokines from macrophages decreases cardiac inflammation (Frangogiannis, 2014). In contrast, macrophage-induced inflammation also mediates the progression of I/R, in which NLRP3 inflammasome activation is a major mechanism inducing inflammation (Nazir et al., 2017; Zhang T et al., 2020).

Farrerol (FA), naturally found in *Rhododendron* dauricum L. leaves, is a dihydro flavonoid active substance with antibacterial, anti-inflammatory, antioxidant, and other biological activities (Li et al., 2013; Ran et al., 2018; Li et al., 2019). Given its strong anti-inflammatory and antioxidant effects, it may have a protective effect against myocardial I/R injury. Therefore, this study explored whether FA might protect the heart against I/R-induced cardiac injury, and clarified the potential mechanism of FA by examining a variety of signaling pathways.

## Materials and Methods

### Reagents

FA (95403-16), adenosine triphosphate (ATP) (A2383), lipopolysaccharide (LPS) (L7770), anti-Flag (F2555), anti-VSV (V4888), and anti-GAPDH (G9545) were purchased from Sigma. Anti-NLPR3 (AG-20B-0014) and anti-Caspase-1 (P20) (AG-20B-0042) were purchased from AdipoGen. Anti-IL-1β (P17) (AF-401-NA) was purchased from R&D Systems. anti-LC3B (A19665), anti-Nrf2 (A1244), anti-pro-Caspase-1 (A16792), anti-Cleaved Caspase-3 (A11021), and anti-pro-IL-1β (A11369) were purchased from Abclonal. anti-Cleaved PARP1 (sc-56196), anti-Bcl2 (sc-7382), anti-Bax (sc-7480), anti-ATG3 (sc-393660), anti-ASC (sc-514414), and anti-NEK7 (sc-393539) were obtained from Santa Cruz Biotechnology.

### Animals and Drug Treatments

The experimental procedure and protocol were approved by the Ethical Use Committee of Shantou University. Wild-type (WT) mice, and *Nrf2* ^*−/−*^ and *Nlrp3* ^*−/−*^ mice in a C57BL/6J background were purchased from GemPharmatech. C57BL/6J mice were administered FA or an equal volume of carrier 7 days before reperfusion. The mice were divided into five groups: 1) Sham group (*n* = 10), this group only received simple skin incision; 2) I/R group (*n* = 10), C57BL/6J mice were injected with normal saline at the same dose for 7 days before I/R; 3) I/R + FA (10 mg/kg/day) group (*n* = 10); 4) I/R + FA (40 mg/kg/day) group (*n* = 10); 5) FA group, C57BL/6J mice were intraperitoneally injected with FA (40 mg/kg/day) for 7 days before I/R.

To explore whether FA might play a protective role in the myocardium through the NRF2 pathway, we divided *Nrlf2* ^*−/−*^ mice into four groups: a WT (I/R) group (*n* = 10), WT (I/R + FA) group (*n* = 10), *Nrlf2* ^*−/−*^ (I/R) group (*n* = 10), and *Nrlf2* ^*−/−*^ (I/R + FA) group (*n* = 10). The FA group was intraperitoneally injected with FA (40 mg/kg/day) for 7 days before I/R, and the other groups were injected with the same volume of normal saline.

To explore whether FA might play a protective role in the myocardium through the autophagy-dependent pathway, we injected 3-methyladenine (3-MA) (Xia et al., 2021) (30 mg/kg) followed by 40 mg/kg FA administered 2 h afterward daily for 7 days.

In the macrophage clearance experiment, mice were pretreated with clodronate liposomes (van Rooijen and van Kesteren-Hendrikx, 2002; Moreno, 2018) (200 μl/mouse) or control liposomes daily, followed by 40 mg/kg of FA for 7 days.

### I/R Model

Myocardial I/R injury models of C57BL/6J, *Nrf2* ^*−/−*^, and *Nlrp3* ^*−/−*^ mice were established as described previously (Zhao et al., 2019). In brief, mice were anesthetized by intraperitoneal injection of pentobarbital sodium at 50 mg/kg. Open the neck first, expose the trachea, and connect the small animal ventilator visually. Needle electrodes were fixed under the skin of the limbs of mice to monitor the changes in limb lead II electrocardiograms. Open the thoracic cavity layer by layer between the third and fourth intercostal space of the left chest. The pericardium was separated, and the heart was exposed. The left anterior descending artery was ligated with silk thread and a PE10 tube for 30 min. After 30 min of ischemia, the PE10 suture was removed. After 24 h of reperfusion, the blood and hearts of mice were collected for testing of various indexes. Mice in the sham operation group underwent the same procedure as those in the model group, but only the silk thread was retained under the left anterior descending artery without ligation or reperfusion.

### Cell Culture and Extraction of Cell Supernatant Protein

Bone marrow cells from the femur and tibia in C57BL/6J mice were collected. Bone marrow-derived macrophages (BMDM) were cultured and differentiated in RPMI-1640 medium containing 10% fetal bovine serum, 2 mM L-glutamine, 1 mM sodium pyruvate, and 50 ng/ml mouse macrophage cluster stimulating factor for 7 days. To extract cell supernatant proteins, cell supernatants were collected and centrifuged at 1,000 g for 5 min. After dead cells were removed, 600 μl of supernatant was carefully transferred to another clean EP tube. An equal volume of pre-cooled methanol and 1/4 volume of chloroform were added and vortexed. The supernatant was aspirated, 500 μl of pre-cooled methanol was added and vortexed, and the tubes were centrifuged at 13,000 r/min for 5 min. The supernatant was carefully removed and discarded. The EP tube loaded with precipitate was placed in a drying chamber at 37°C for 5 min and dissolved in 30 μl 2.5× loading buffer. After boiling at 95°C for 5 min, samples were loaded for western blot analysis.

### H&E Staining

The heart tissues from each group of mice were fixed with 4% paraformaldehyde overnight at 4°C and embedded in paraffin. The paraffin was sliced 5 μm thick and incubated with hematoxylin and eosin (H&E) with an H&E staining kit (C0105S, Beyotime, China) according to the manufacturer’s protocol.

### Enzyme-Linked Immunosorbent Assay

The expression of CK-MB (MU30025, Bioswamp, China), LDH (MU30023, Bioswamp, China), troponin-1 (MU30421, Bioswamp, China), NT-proBNP (MU30252, Bioswamp, China), IL-1β (MU30369, Bioswamp, China), IL-6 (MU30044, Bioswamp, China), and TNF-α (MU30030, Bioswamp, China) in cells, tissues, or serum samples was determined with an ELISA kit according to the kit instructions.

### RT-qPCR

The relative expression of *Il-b*, *Il-6*, *Tnfa*, and *F4/80* in cells and tissues was detected by RT-qPCR. TRIzol reagent was used to extract total RNA from cells and tissues. First-strand cDNA was generated with a Reverse Transcription System Kit (Thermo Fisher Scientific), and RT-qPCR was performed with an SYBR Green PCR kit (Thermo Fisher Scientific) according to the manufacturer’s instructions. All qRT-PCR was performed on a Biosystems 7300 Real-Time PCR system (Thermo Fisher Scientific). Relative gene expression levels were calculated with the 2^−ΔΔCt^ method. The primer sequences are shown in Table 1.

**TABLE 1:** Primer for real-time RT-PCR was used in this study.

Gene	Sequences	Species
*Il-1b*	Forward: CAC​AGC​AGC​ACA​TCA​ACA​AG	Mouse
	Reverse: GTG​CTC​ATG​TCC​TCA​TCC​TG	
*Il-6*	Forward: CTC​TGG​GAA​ATC​GTG​GAA​AT	Mouse
	Reverse: CCA​GTT​TGG​TAG​CAT​CCA​TC	
*Tnfa*	Forward: GAC​GTG​GAA​CTG​GCA​GAA​GAG	Mouse
	Reverse: TTG​GTG​GTT​TGT​GAG​TGT​GAG	
*Gapdh*	Forward: AGG​TCG​GTG​TGA​ACG​GAT​TTG	Mouse
	Reverse: TGT​AGA​CCA​TGT​AGT​TGA​GGT​CA	

### Assessment of Oxidative Stress in Heart Tissue

Mice in each experimental group were euthanized, and the hearts were removed and washed retrogradely with Krebs-Ringer solution maintained at 37°C. The hearts were then weighed, and the cardiac tissue was homogenized on ice in chilled phosphate-buffered saline (PBS) containing 1 mM EDTA. Centrifuge at 7,000 rpm/min for 10 min. Measure the protein concentration of the supernatant. The supernatant was used to analyze lipid peroxidation levels and glutathione peroxidase (GSH-Px), superoxide dismutase (SOD), and malondialdehyde (MDA) levels. The vascular nicotinamide adenine dinucleotide phosphate (NADPH) oxidase is a major source of ROS and the NOX4 is the major NAD(P)H oxidase isoform in cardiomyocytes, so we use Western blotting to detect the expression level of NOX4.

### Western Blot Analysis

Total protein was isolated from tissues and cells with 1× radioimmunoprecipitation assay (RIPA) buffer containing phosphatase and protease inhibitors. After protein concentrations were determined, samples were separated by SDS-PAGE and transferred to a PVDF membrane. The membrane was blocked in Tris-buffered saline-Tween 20 (TBST) buffer containing 5% BSA for 1 h, and incubated with primary antibody (anti-Flag (1:500), anti-VSV (1:1,000), anti-GAPDH (1:10,000), Anti-NLPR3 (1:500), Anti-caspase-1 (P20) (1:1,000), Anti-IL-1β (P17) (1:1,000), Anti-LC3B (1:1,000), anti-Nrf2 (1:1,000), Anti-pro-caspase-1 (1:1,000), anti-pro-interleukin-1 β (pro-IL-1β) (1:1,000), Anti-PARP1 (1:1,000), anti-Bcl2 (1:1,000), anti-Bax (1:1,000), anti-ATG3 (1:1,000), anti-ASC (1:1,000), Anti-caspase-1 (1:1,000), and anti-NEK7 (1:1,000).) at 4°C overnight. After being washed with TBST buffer three times, membranes were incubated with HRP labeled mouse or rabbit secondary antibody (1:5,000 diluted) at room temperature for 1.5 h. They were then incubated on a shaker for 1 h. The membranes were then washed with TBST five times for 5 min each. Chemiluminescence reagent was used for luminescence development. The analysis was performed with Quantity One 4.4 software.

### Immunoprecipitation

For endogenous interaction, BMDM cells in each experimental group were lysed in NP-40 buffer mixed with protease inhibitors and centrifuged at 4°C for 15 min. The cell lysates were collected and incubated with IP antibodies [NEK7 (1:100) or ASC (1:100)] overnight at 4°C. Protein A/G beads were added and incubated for 4 h at 4°C. Protein A/G beads pulled down the target protein for detection using Western blotting. For exogenous interaction, HEK-293T cells were transfected with Lipofectamine 2000 according to the kit instructions. After 24 h, cells were collected and lysed in NP-40 buffer mixed with protease inhibitors. Cell lysates were immunoprecipitated with the anti-Flag agarose gels (1:100, Sigma, A2220) and detected using Western blotting.

### Statistical Analysis

Results are shown as mean ± standard deviation from three independent experiments or are representative of three independent experiments. SPSS19.0 software was used for statistical analysis. For most experiments, differences between the two groups were assessed using two-tailed unpaired Student’s t-tests and one-way analysis of variance. Non-parametric Mann-Whitney tests were used to compare the differences between more than two groups when the variances were significantly different. If a *p*-value was <0.05, the difference was considered statistically significant.

## Results

### FA Has a Protective Effect Against I/R-Induced Myocardial Injury in Mice

**FIGURE 1:**
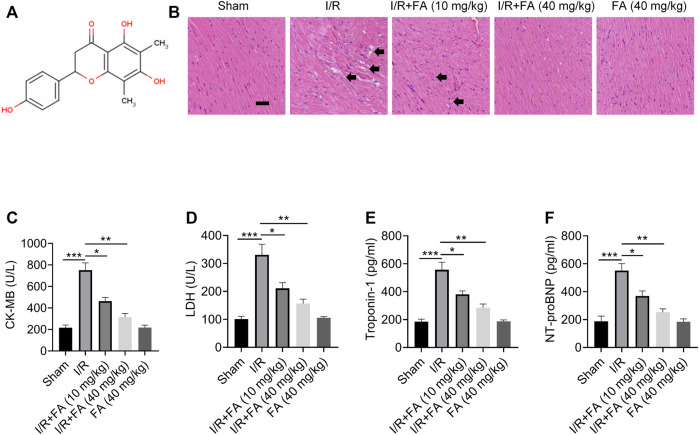
The protective effect of FA against I/R-induced myocardial injury in mice. **(A)** Chemical structural formula of FA. **(B)** HE staining results of heart tissue sections in each experimental group of WT mice. **(C–F)** Expression of CK-MB, LDH, troponin-1, and NT-proBNP in the serum in each experimental group of WT mice, detected by ELISA. Data are expressed as the means ± sd from three independent experiments. ^*^ *p* < 0.05, ^**^ *p* < 0.01, and ^***^ *p* < 0.001.

After the successful establishment of the mouse model, treatment with FA (structure in Figure 1A) was performed. HE staining results indicated significant injury in the I/R group, whereas the I/R + FA (10 mg/kg or 40 mg/kg) treatment group showed significantly less histological injury caused by I/R (Figure 1B). In addition, FA significantly decreased the expression of CK-MB, LDH, troponin-1, and NT-proBNP induced by I/R, and FA alone treatment group did not affect the heart, immune system, and other tissue functions in healthy mice (Figures 1C–F). Therefore, FA effectively protects against I/R-induced myocardial injury.

### FA Alleviates the Inflammatory Response to I/R-Induced Myocardial Injury in Mice

**FIGURE 2:**
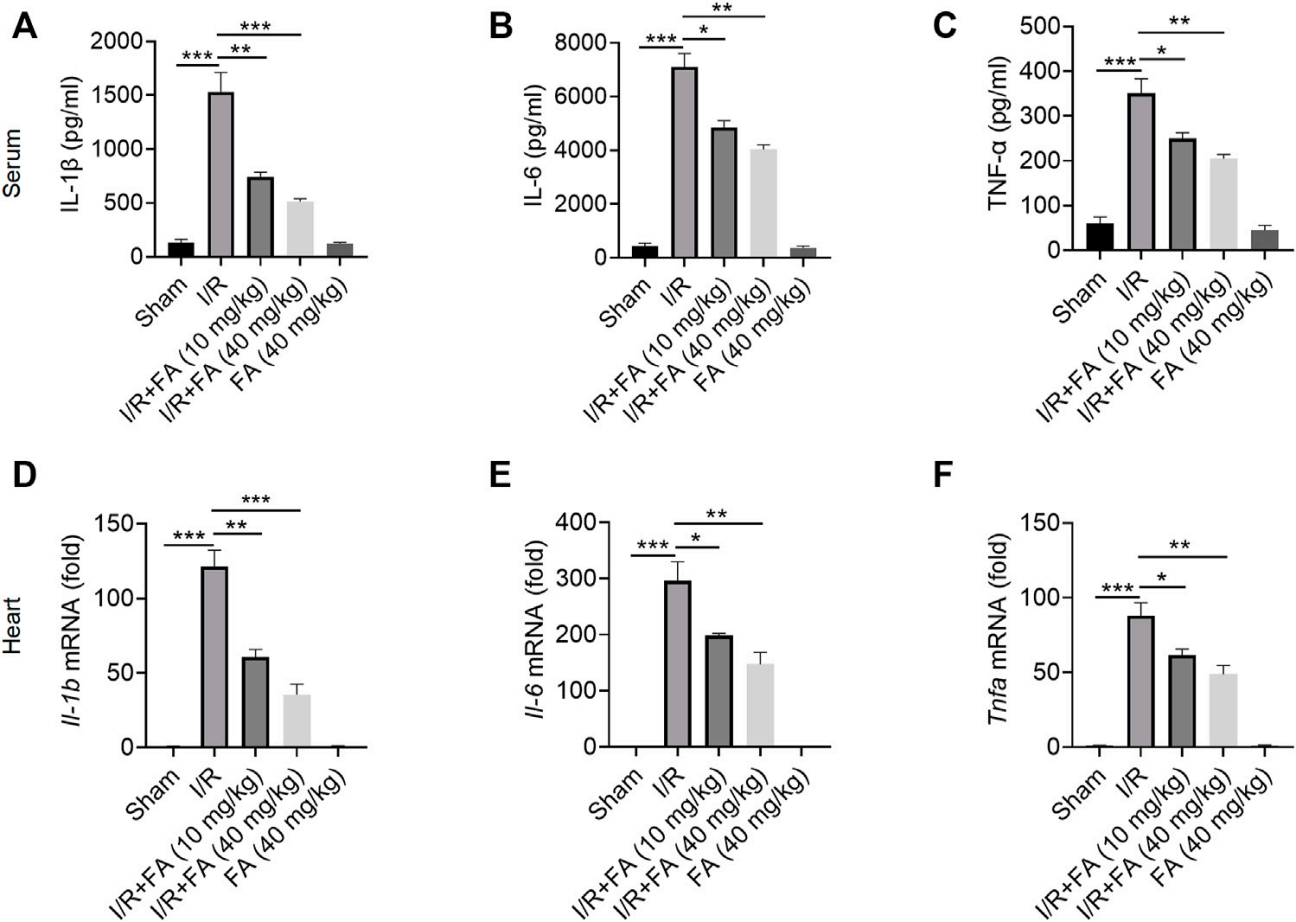
FA alleviates the inflammatory response to I/R-induced myocardial injury in mice. **(A–C)** Expression of the inflammatory factors IL-1β, IL-6, and TNF-α in WT mice was detected by ELISA. **(D–F)** mRNA expression of the inflammatory cytokines *Il-1b*, *Il-6*, *Il-17*, and *Tnfa* in heart tissue of WT mice in each experimental group, detected by RT-PCR. Data are expressed as the means ± sd from three independent experiments. ^*^ *p* < 0.05, ^**^ *p* < 0.01, and ^***^ *p* < 0.001.

As shown in Figure 2, I/R treatment increased the expression of IL-1β, IL-6, and TNF-α in the serum, whereas FA treatment decreased the expression of these inflammatory factors (Figure 2A–C). In addition, the RT-qPCR I/R treatment significantly upregulated the mRNA levels of these inflammatory factors in the heart tissues, while FA treatment significantly decreased their expression (Figures 2D–F). Thus, FA alleviates the inflammatory response that leads to myocardial injury in mice after I/R.

### FA Alleviate IR I/R-Induced Oxidative Stress in Mice

**FIGURE 3:**
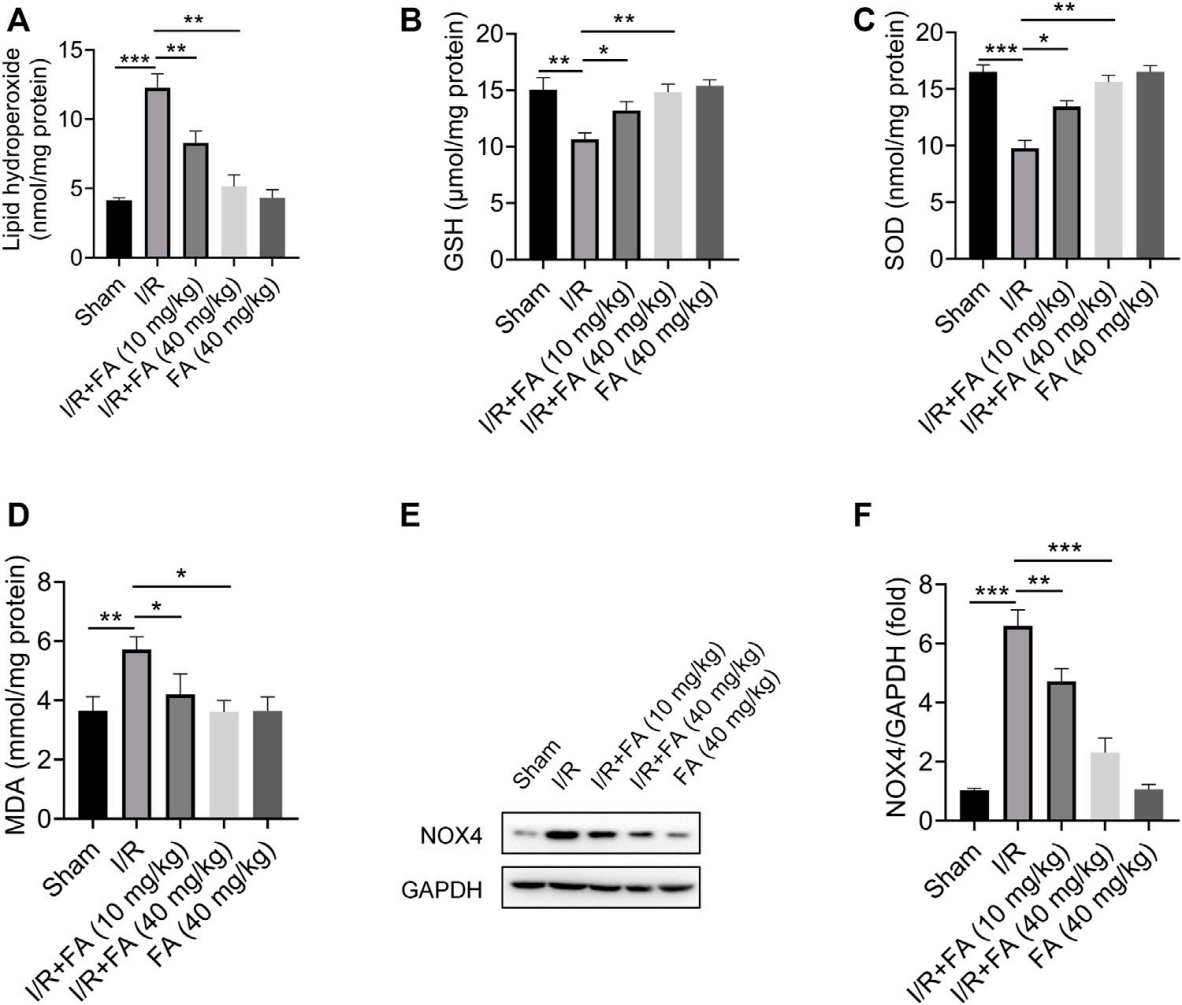
Effect of FA on oxidative stress in the cardiac tissue homogenate. **(A)** Lipid hydroperoxide, **(B)** glutathione peroxidase, **(C)** superoxide dismutase, **(D)** malondialdehyde, **(E–F)** Western blotting analysis of NOX4 in the mice myocardium. Data are expressed as the mean ± SD from three independent experiments or are representative of three independent experiments. ^*^ *p* < 0.05, ^**^ *p* < 0.01, and ^***^ *p* < 0.001.

The results in Figure 3 showed that Lipid hydroperoxide concentration and MDA level were significantly higher in the IR group than those in the sham group and IR + FA group (*p* < 0.05), whereas the levels of SOD and GSH-Px were significantly lower in the IR group compared with IR + FA group. Furthermore, the NOX4 expression was significantly decreased in the IR + FA group when compared with the IR group. We did not find significant changes in these antioxidant enzymes and reductive oxidation products between the sham-operated group and the FA-only group. Therefore, these data indicate that FA can alleviate oxidative stress caused by IR.

### FA Inhibits I/R-Induced Apoptosis in Mice

**FIGURE 4:**
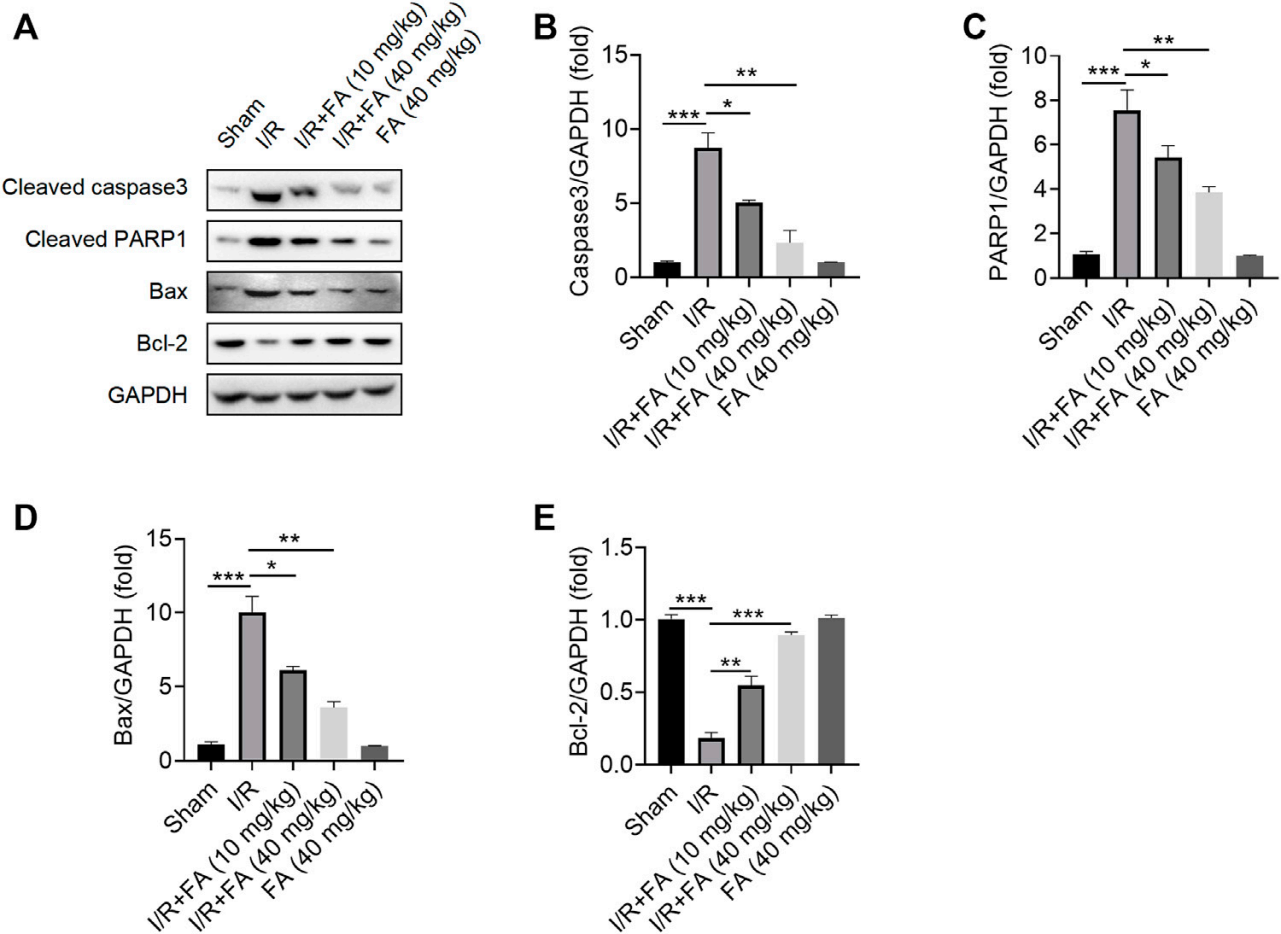
FA inhibits I/R-induced apoptosis in mice. **(A–E)** Western blotting detection of the expression of the apoptosis-associated proteins cleaved PARP1, cleaved caspase-3, Bcl-2, and Bax in WT mice in each experimental group. Data are expressed as the means ± sd and are representative of three independent experiments. ^*^ *p* < 0.05, ^**^ *p* < 0.01, and ^***^ *p* < 0.001.

The results showed that FA significantly decreased the protein expression of cleaved CASP3, cleaved PARP1 and Bax while increasing Bcl-2 expression in the heart tissues (Figure 4A–E). Therefore, FA alleviates I/R-induced apoptosis in I/R mice.

### FA Increases Nrf2 Expression, but Nrf2 Knockout Does not Eliminate the Myocardial Protective Effect of FA

**FIGURE 5:**
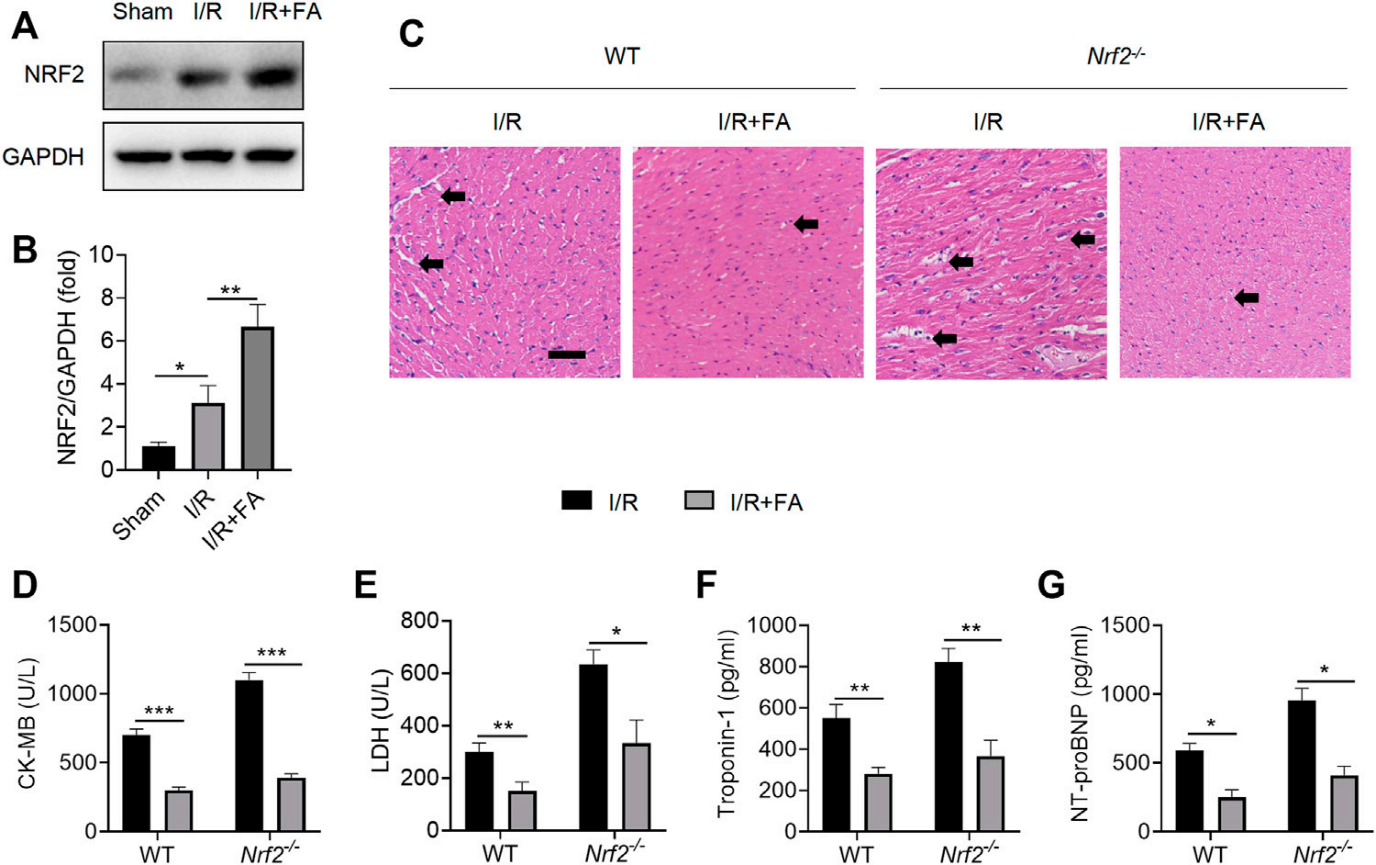
FA induces an increase in NRF2, but NRF2 knockout does not eliminate the myocardial protective effect of FA. **(A,B)** The expression of NRF2 protein in each group of WT mice was detected by western blotting. **(C)** HE staining results of heart tissue sections in WT mice and *Nrf2* ^*−/−*^ mice in each experimental group. **(D–G)** Expression of CK-MB, LDH, troponin-1, and NT-proBNP in the serum of each experimental group in WT mice and *Nrf2* ^*−/−*^ *mice*. Data are expressed as the means ± sd from three independent experiments or are representative of three independent experiments. ^*^ *p* < 0.05, ^**^ *p* < 0.01, and ^***^ *p* < 0.001.

To further determine whether the protective effect of FA against myocardial injury NRF2-dependent, we used *Nrf2* ^*−/−*^ mice. FA significantly increased NRF2 expression levels in I/R-treated WT mice in the heart tissues (Figure 5A,B). However, in *Nrf2* ^−/−^ mice, HE staining indicated that the protective effect of FA was unaffected, and the I/R-induced cardiac tissue damage was more severe in Nrf2−/− mice than in WT mice (Figure 5C). Furthermore, FA significantly decreased the increases in CK-MB, LDH, troponin-1, and NT-proBNP in the serum (Figure 5D–G). These data suggest that the presence of NRF2 does not affect the protective effect of FA against I/R-induced myocardial injury, but the increase in NRF2 warrants further exploration.

### FA Does Not Alleviate I/R-Induced Injury Through Autophagy

**FIGURE 6:**
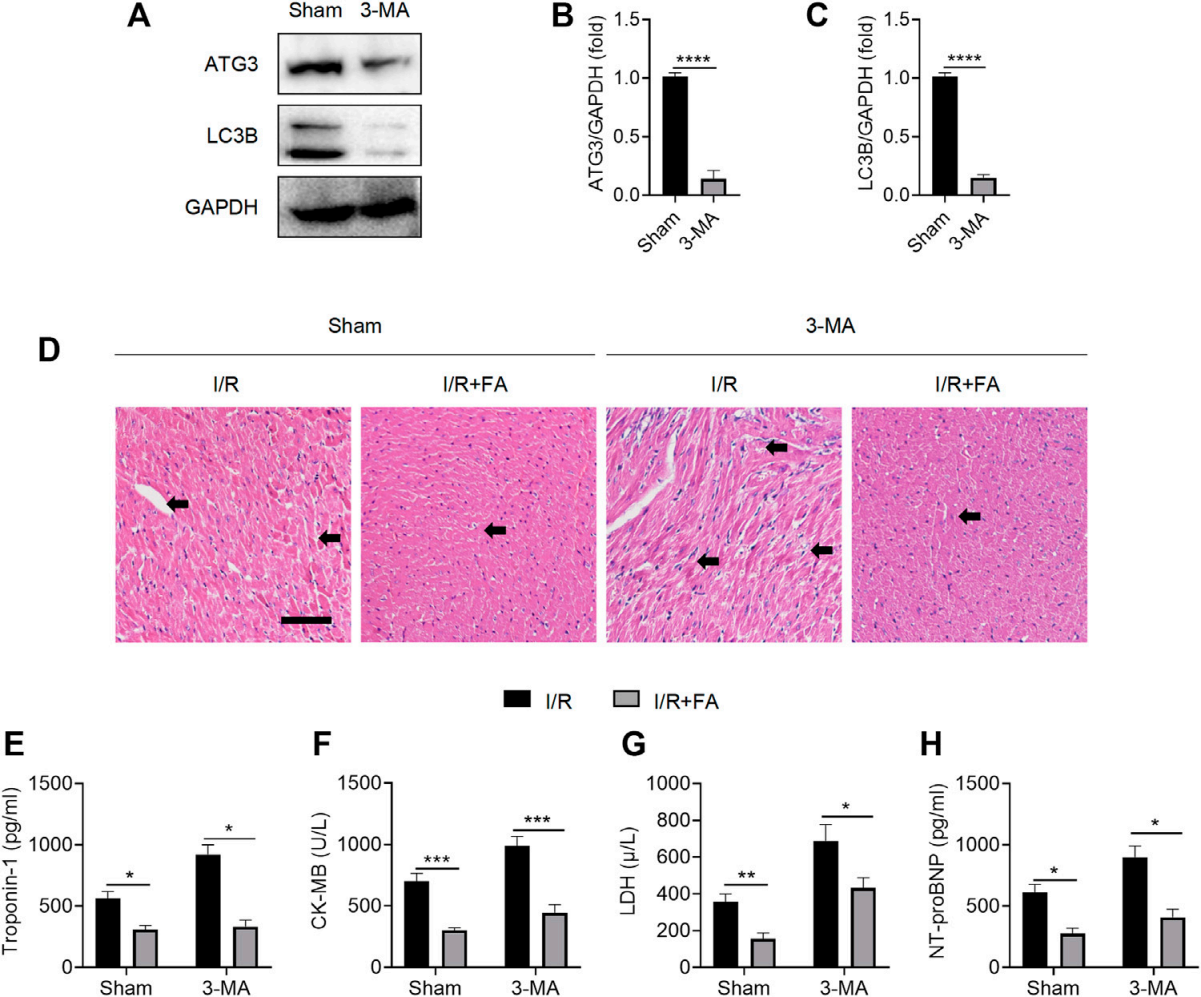
FA does not alleviate I/R-induced myocardial injury through autophagy. **(A–C)** Western blotting detection of the expression of ATG3 and LC3B proteins in the heart tissue of WT mice treated with saline or 3-MA. **(D)** HE staining results of heart tissue sections in WT mice treated with saline or 3-MA. **(E–H)** Expression of CK-MB, LDH, troponin-1, and NT-proBNP in the serum of each experimental group, detected by ELISA. Data are expressed as the means ± sd from three independent experiments or are representative of three independent experiments. ^*^ *p* < 0.05, ^**^ *p* < 0.01, ^***^ *p* < 0.001, and ^****^ *p* < 0.0001.

Protective autophagy has been found to alleviate I/R-induced myocardial injury (Li et al., 2020). The widely used autophagy inhibitor 3-MA significantly inhibited the expression of autophagy marker ATG3 and decreased the ratio of the autophagy marker LC3II/LC3I at the experimental dose (Figure 6A–C). As shown in Figure 6D, after autophagy was inhibited by 3-MA, HE staining indicated that FA still alleviated I/R-induced injury. In addition, FA significantly decreased the increases in CK-MB, LDH, troponin-1, and NT-proBNP in the serum (Figure 6D–H). Accordingly, FA does not alleviate I/R-induced injury by inducing autophagy.

### Macrophage Clearance Inhibits the Myocardial Protective Effect of FA

**FIGURE 7:**
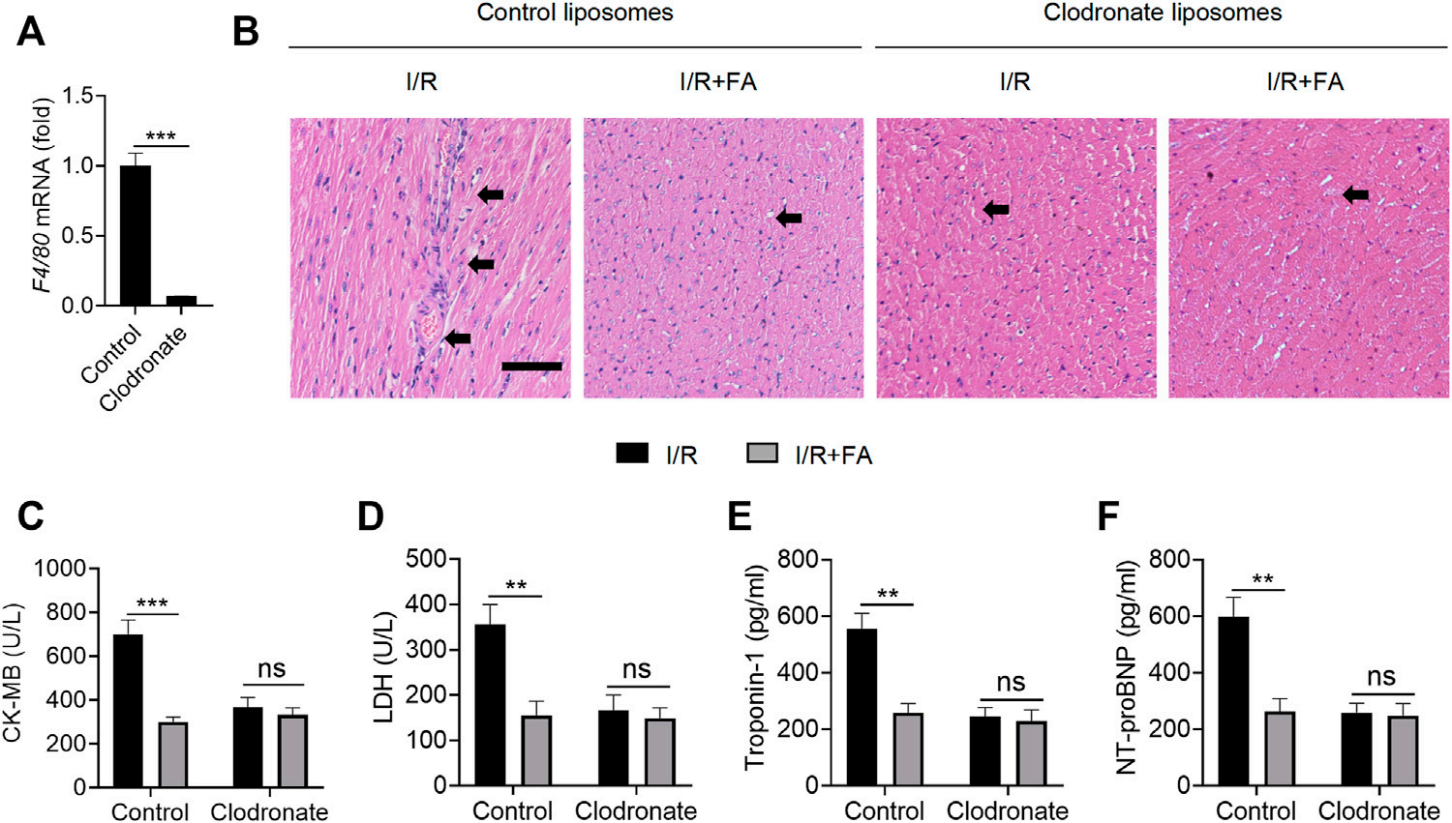
Macrophage clearance inhibits the myocardial protective effect of FA. **(A)** The mRNA expression of F4/80 in the heart tissue of WT mice treated with clodronate liposomes was detected by RT-PCR. **(B)** HE staining of heart tissue sections in each experimental group after treatment with clodronate liposomes or control liposomes. **(C–F)** Expression of CK-MB, LDH, troponin-1, and NT-proBNP in the serum of each experimental group after treatment with clodronate liposomes and control liposomes. Data are expressed as the means ± sd from three independent experiments. ^**^ *p* < 0.01, ^***^ *p* < 0.001; ns indicates no significance.

Immune inflammation is an important factor in I/R induced myocardial injury, and macrophages are the main infiltrating inflammatory cells acting after myocardial injury (Vilahur and Badimon, 2014). FA might exert protective effects in the myocardium by modulating macrophage-mediated inflammation. To test this hypothesis, we used clodronate liposomes to remove macrophages. F4/80 (Austyn and Gordon, 1981; van den Berg and Kraal, 2005) mRNA in clodronate-treated mouse hearts was significantly lower than in control liposomes (Figure 7A), thus suggesting that clodronate effectively depleted macrophages. In mice injected with liposomes in the control group, FA alleviated the I/R-induced increases in the CK-MB, LDH, troponin-1, and NT-proBNP levels in the serum. After injection of clodronate, HE staining indicated that FA was no longer protected in the myocardium (Figure 7B). We observed no significant changes in CK-MB, LDH, troponin-1, and NT-proBNP in the FA group compared to the control group in the serum (Figure 7C–F). Therefore, the protective effect of FA against I/R-induced myocardial injury may rely on its targeting of macrophages.

### FA Inhibits I/R-Induced Myocardial Injury by Inhibiting NLRP3 Inflammasome Activation

**FIGURE 8:**
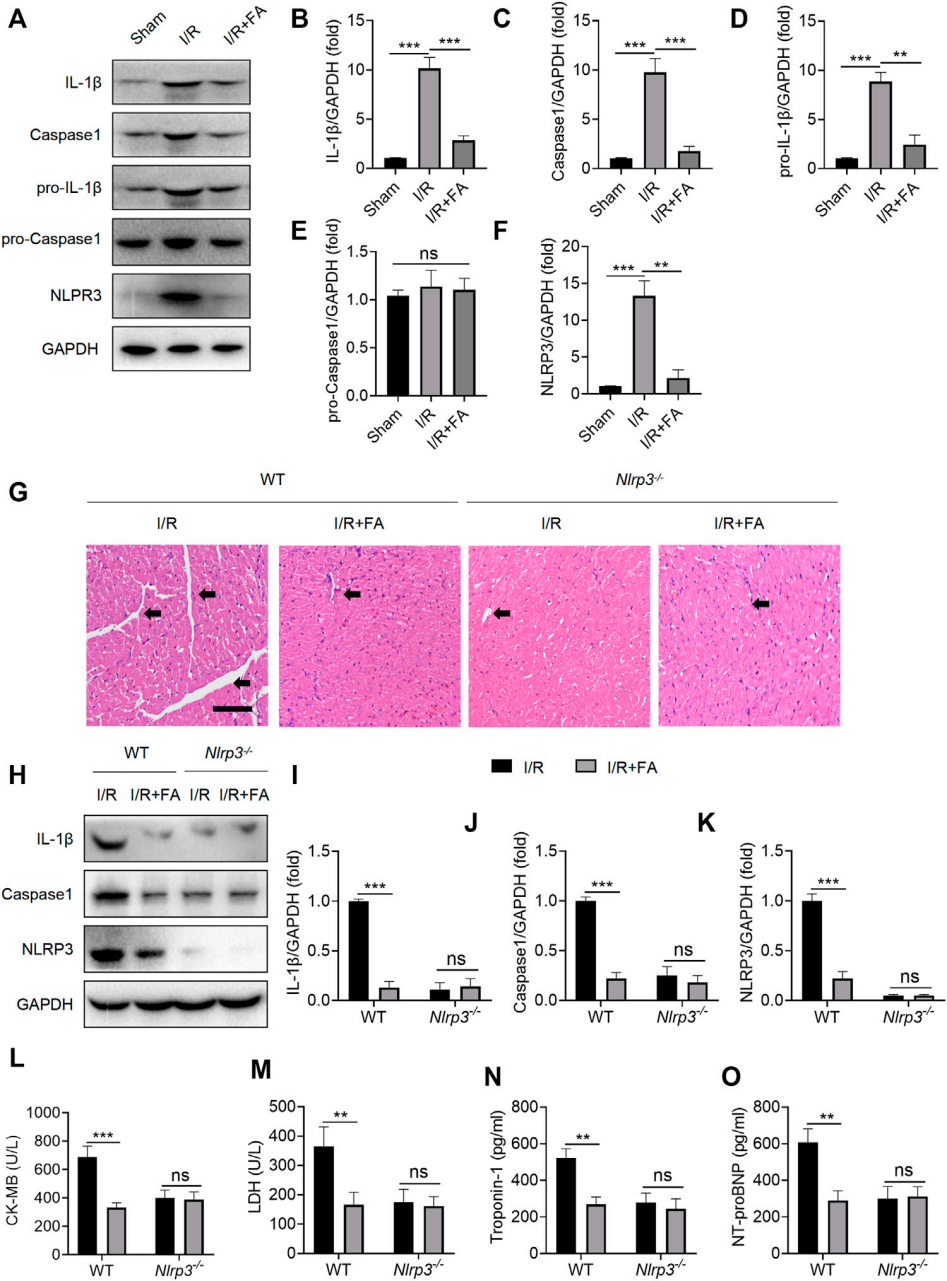
FA inhibits I/R-induced myocardial injury by inhibiting NLRP3 inflammasome activation. **(A–F)** Expression of NLRP3, pro-caspase-1, caspase-1, pro-IL-1β, and IL-1β in the heart tissue of WT mice was detected by western blotting. **(G)** HE staining of heart tissue sections in WT mice and *Nlrp3* ^*−/−*^mice. **(H–K)** Expression of IL-1β, caspase-1, and NLRP3 in heart tissues of WT mice and *Nlrp3* ^*−/−*^ mice in each experimental group, detected by western blotting. **(L–O)** Expression of CK-MB, LDH, troponin-1, and NT-proBNP in the serum of each experimental group in WT mice and *Nlrp3* ^*−/−*^mice, determined by ELISA. Data are expressed as the means ± sd from three independent experiments or are representative of three independent experiments. ^**^ *p* < 0.01, ^***^ *p* < 0.001; ns indicates no significance.

According to prior reports (Lopez-Castejon and Brough, 2011; Maruyama et al., 2019), activation of inflammasomes in macrophages is a key pathway for IL-1β secretion. Moreover, NLRP3 inflammasome activation is an important pathological factor leading to I/R myocardial injury. First, we examined whether FA might affect NLRP3 inflammasome activation in WT I/R mice. FA significantly decreased the protein expression of NLRP3, caspase-1, IL-1β, and pro-IL-1β in WT mice, but did not significantly alter levels of the pro-caspase-1 protein (Figures 8A–F). However, in *Nlrp3* ^−/−^ mice, the protective effect of FA in the myocardium was eliminated (Figure 8G). Meanwhile, in NLRP3 knockout mice, FA did not affect the expression of IL-1β and caspase-1 (Figures 8H–K), thus, suggesting that FA affects NLRP3 inflammasome activation. Furthermore, in *Nlrp3* ^−/−^ mice, FA did not inhibit the levels of CK-MB, LDH, troponin-1, and NT-proBNP in the serum (Figures 8L–O). Therefore, these results suggest that FA protects against I/R-induced myocardial injury by inhibiting NLRP3 inflammasome activation.

### FA Inhibits NLRP3 Inflammasome Activation by Interfering With NLRP3 Binding to NEK7 in Macrophages

**FIGURE 9:**
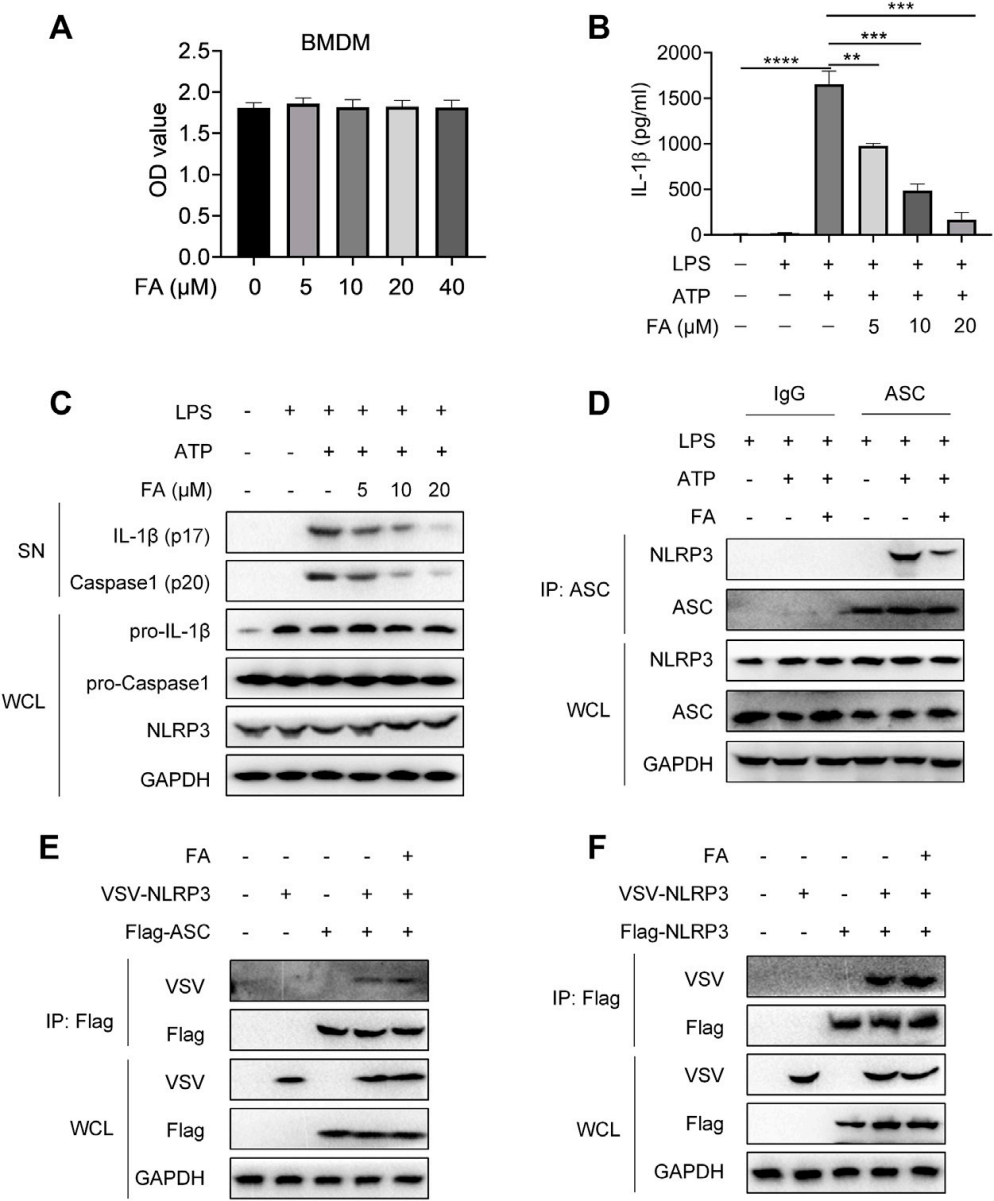
FA does not affect the interaction between NLRP3 and ASC or NLRP3. LPS-primed BMDMs were treated with various concentrations of FA (20 μM) for 15 min and then stimulated with ATP for 30 min. **(A)** Expression of IL-1 β in each group after treatment with different concentrations of FA, detected by ELISA. **(B)** Western blotting detection of the expression of IL-1β and caspase-1 (P20) in cell supernatants (SN), and pro-IL-1β and pro-caspase-1 in total protein. **(C)** CCK-8 detection of the effects of different concentrations of FA on the viability of BMDMs. **(D)** Endogenous IP with ASC antibody or IgG, performed in LPS-primed BMDMs stimulated with ATP in the presence or absence of FA (20 μM). **(E)** IP and western blot analysis of NLRP3 and ASC interaction in the presence or absence of FA (20 μM) in HEK-293T cells. **(F)** IP and western blot analysis of NLRP3 and NLRP3 interaction in the presence or absence of FA (20 μM) in HEK-293T cells. Data from three independent experiments or are representative of three independent experiments.

**FIGURE 10:**
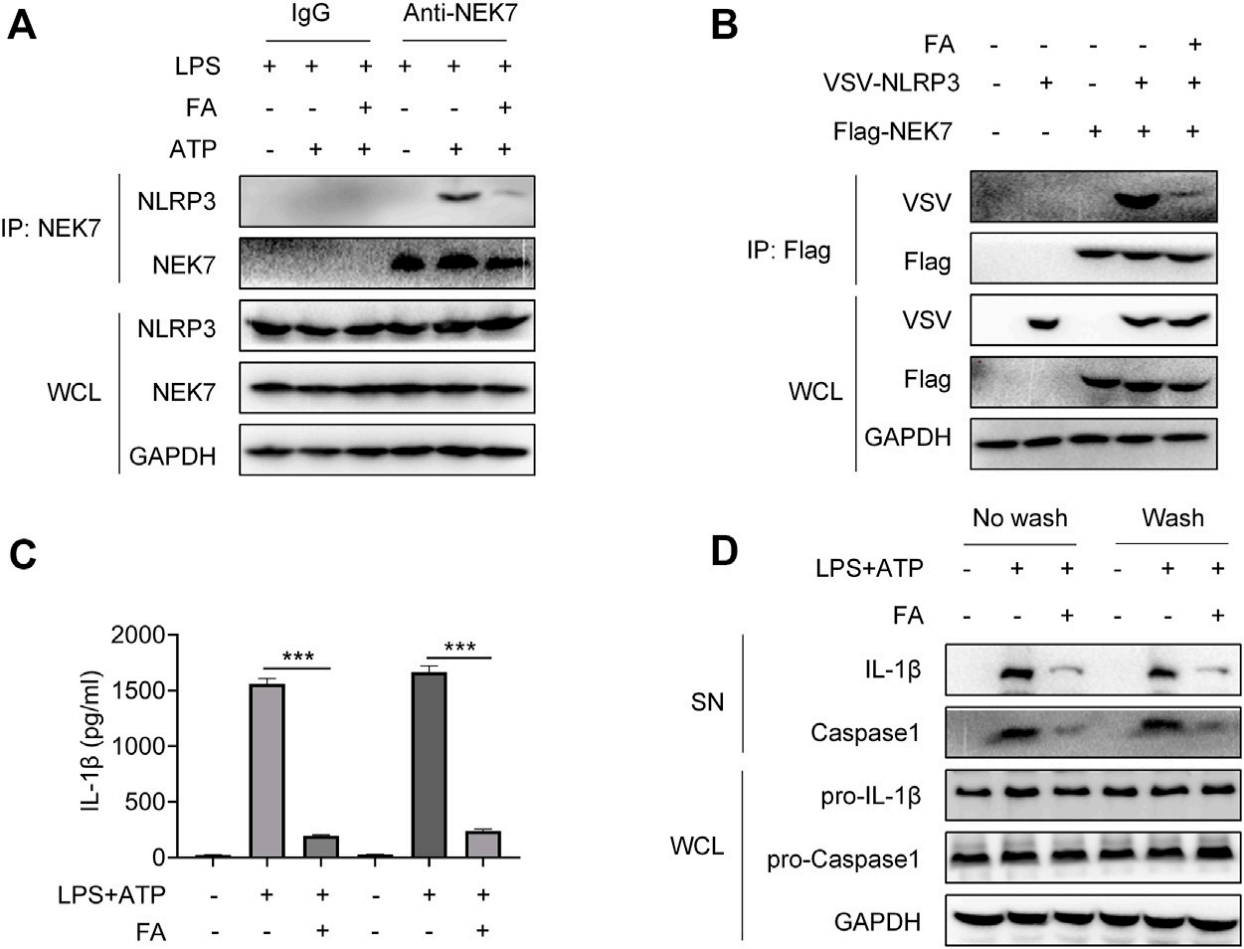
FA inhibits NLRP3 inflammasome activation by interfering with NLRP3 and NEK7. LPS-primed BMDMs were treated with various concentrations of FA (20 μM) for 15 min and then stimulated with ATP for 30 min. **(A)** Endogenous immunoprecipitation (IP) with NEK7 antibody or IgG in LPS-primed BMDMs stimulated with ATP in the presence or absence of FA (20 μM). **(B)** IP and western blot analysis of NEK7-NLRP3 interaction in HEK-293T cells treated with FA (20 μM). LPS-primed BMDMs were treated with FA (20 μM) for 15 min, washed three times, and then stimulated with ATP for 30 min. ELISA analysis of IL-1β in supernatants in each group in the presence or absence of FA (20 μM) **(C)**, and western blot analysis of cleaved IL-1β and caspase-1 (p20) in SN, and pro-IL-1β and pro-caspase-1 in the whole-cell lyses (WCL) **(D)**. Data are expressed as the means ± sd from three independent experiments or are representative of three independent experiments. **p < 0.01, ***p < 0.001, and ****p < 0.0001.

Next, we investigated whether FA might inhibit NLRP3 inflammasome activation in macrophages. First, we examined the effect of FA on macrophage toxicity. The CCK8 results indicated that FA (0–40 μM) did not affect the proliferation of BMDM (Figure 9A). According to ELISA, FA decreased the release of IL-1β in BMDM (Figure 9B). Western blot results indicated that FA also inhibited the expression of IL-1β and caspase-1 in cell supernatants (Figure 9C), thus suggesting that FA effectively inhibits NLRP3 inflammasome activation in macrophages. Endogenous IP experiments indicated that FA inhibited NLRP3 and ASC binding in macrophages (Figure 9D). Unexpectedly, exogenous IP revealed that FA did not affect NLRP3 interaction with ASC in HEK-293T cells (Figure 9E), thus, suggesting that FA does not inhibit NLRP3 inflammasome activation by affecting NLRP3’s interaction with ASC. Furthermore, FA did not influence the NLRP3-NLRP3 interaction (Figure 9F). The interaction between NEK7 and NLRP3 plays a key role in ASC recruitment and NLRP3 oligomerization. We then studied the effect of FA on the interaction between NLRP3 and NEK7. Based on endogenous IP, FA inhibited the NEK7-NLRP3 interaction in BMDM (Figure 10A). In agreement with this finding, exogenous IP results also indicated that FA directly interfered with NEK7-NLRP3 interaction (Figure 10B). To study the reversibility of the interaction between FA and NLRP3, we analyzed the binding properties of FA and NLRP3. Washing experiments revealed that FA inhibited ATP-induced IL-1β secretion and caspase-1 activation after washing, thus suggesting that the inhibition of FA was irreversible (Figure 10C,D). Together, these results suggested that FA inhibits the activation of the NLRP3 inflammasome by blocking the NEK7-NLRP3 interaction.

## Discussion

Despite significant advances in technology and drugs for the treatment of CVD, the disease remains the leading cause of death worldwide, with coronary heart disease being a key factor (Mokhtari-Zaer et al., 2018; Silvis et al., 2021). After acute myocardial infarction, the blood flow to the ischemic myocardium must be rapidly restored, but this restoration may lead to additional complications and the aggravation of myocardial injury, known as myocardial I/R injury (Mehta et al., 2016). Therefore, exploring how to decrease the myocardial injury caused by I/R may have high therapeutic value.

FA, found to be naturally present in *Rhododendron* dauricum L*.* leaves, is a natural dihydro flavonoid active substance with antibacterial, anti-inflammatory, antioxidant, and other biological activities (Li et al., 2013; Ran et al., 2018; Li et al., 2019; Qin et al., 2022). In this study, we aimed to find its role and mechanism in I/R-induced injury.

Autophagy is widespread in normal cells, acting as scavengers to meet the metabolic needs of cells and enable the renewal of some organelles (Kamynina et al., 2021). When tissues and cells are injured by various physical and chemical factors, the autophagic lysosomes increase greatly and protect cell injury (Ichimiya et al., 2020). In myocardial I/R, many immune cells and myocardial cells are injured and die, inducing autophagy to remove these dead cells and protect the myocardium (Tschöpe et al., 2021). A previous study has reported that FA promotes autophagy, and thus, protects the liver (Wang et al., 2019). Therefore, we hypothesized that the cardiac protective effect of FA might be associated with autophagy. Here, we blocked autophagy using the widely used autophagy inhibitor 3-MA. Unexpectedly, 3-MA pretreatment did not eliminate the myocardial protective effect of FA, thus, suggesting that FA does not play a protective role in the myocardium through the autophagy-dependent pathway. The protective mechanism of FA in the heart differs from that in liver disease.

The elevated production of reactive oxygen species plays an important role during myocardial I/R injury (Cadenas, 2018). NRF2, a transcription factor, induces gene expression of a range of antioxidant enzymes, such as heme oxygenase-1 (HO-1), superoxide dismutase (SOD), glutathione catalase (GPx), NAD(P)H: quinone oxidoreductase 1 (NQO1), and γ -glutamylcysteine synthase (γ-GCS) to combat oxidative stress (Harris and DeNicola, 2020). Previous studies have shown that FA activates NRF2 by protecting a variety of cells (Cui et al., 2019; Wang et al., 2019; Harris and DeNicola, 2020). Therefore, we used *Nrf2* ^−/−^ mice to explore whether the cardiac protective effects of FA might be associated with the NRF2 pathway. In an *Nrf2* ^−/−^ mouse I/R model, FA still exerted a protective effect in the myocardium. In addition, FA could alleviate excessive oxidative stress by elevating the level of antioxidant enzymes and reducing oxidation products in the cardiac tissue. These data suggest that NRF2 is not a key target for cardioprotection by FA.

Since FA still protected against I/R-induced myocardial injury in *Nrf2* ^−/−^ mice and WT mice with autophagy inhibition, FA does not appear to play a protective role in the myocardium through affecting NRF2 pathways and autophagy. Given that immune-mediated inflammatory responses play important roles in myocardial I/R injury, we hypothesized that FA might affect myocardial cell function by directly regulating immune cells. Because macrophages have important functions as a key factor in myocardial injury (Fan et al., 2019; Peet et al., 2020), we wondered whether FA’s protective role might rely on its action on macrophages. To test this hypothesis, we used clodronate liposomes to deplete macrophages to verify that the cardioprotective effects of FA were associated with macrophages. In control mice injected with liposomes, FA alleviated I/R-induced myocardial injury, whereas the protective effect of FA was weakened after chlorophosphate injection. Moreover, FA did not decrease the increases in CK-MB, LDH, troponin-1, and NT-proBNP in the serum. Therefore, we concluded that FA alleviates I/R-induced myocardial injury by targeting macrophages. However, how does FA act on macrophages?

I/R-induced myocardial injury would produce a large amount of metabolin, which triggers NLRP3 inflammasome activation (Qiu et al., 2017; Zhang J et al., 2020). When inflammatory cells infiltrate the heart, numerous NLRP3 inflammasome spots appear in macrophages (Nazir et al., 2017; Dai et al., 2020). Might FA play a protective role by inhibiting NLRP3 inflammasome in macrophages? We tested this hypothesis by examining the expression of related factors after NLRP3 inflammasome activation. NLRP3 inflammasomes regulate caspase-1 activation and promote the maturation and secretion of IL-1β during the natural immune defense (Burke et al., 2021); they also regulate caspase-1-dependent programmed apoptosis and induce cell death under inflammatory and stressful pathological conditions (Swanson et al., 2019). As shown in Figure 8, we concluded that FA protects against I/R injury by inhibiting NLRP3 inflammasomes in macrophages. Which part of the NLRP3 inflammasome does FA act on?

The NLRP3 inflammasomes are composed of NLRP3, ASC, and caspase-1. When cells are stimulated, NLRP3 is activated and recruited pro-caspase-1 by binding ASCs and forming NLRP3 inflammasomes (Swanson et al., 2019). In response to NLRP3 inflammasomes, pro-caspase-1 is activated and forms caspase-1, which leads to the maturation and secretion of IL-1β, as well as a series of inflammatory responses (He et al., 2016; Shrivastava et al., 2016). NEK7 has been found to be involved in the formation and activation of the NLRP3 inflammasome, and the deletion of NEK7 specifically blocks NLRP3 inflammasome activation (He et al., 2016). Therefore, we tested the expression of these proteins to provide further verification. FA blocked the assembly of NLRP3 inflammasomes by interfering with the interaction of NEK7 and NLRP3 but did not affect the interaction of NLRP3 with ASC and NLRP3. Thus, FA inhibits NLRP3 inflammasome activation.

**FIGURE 11:**
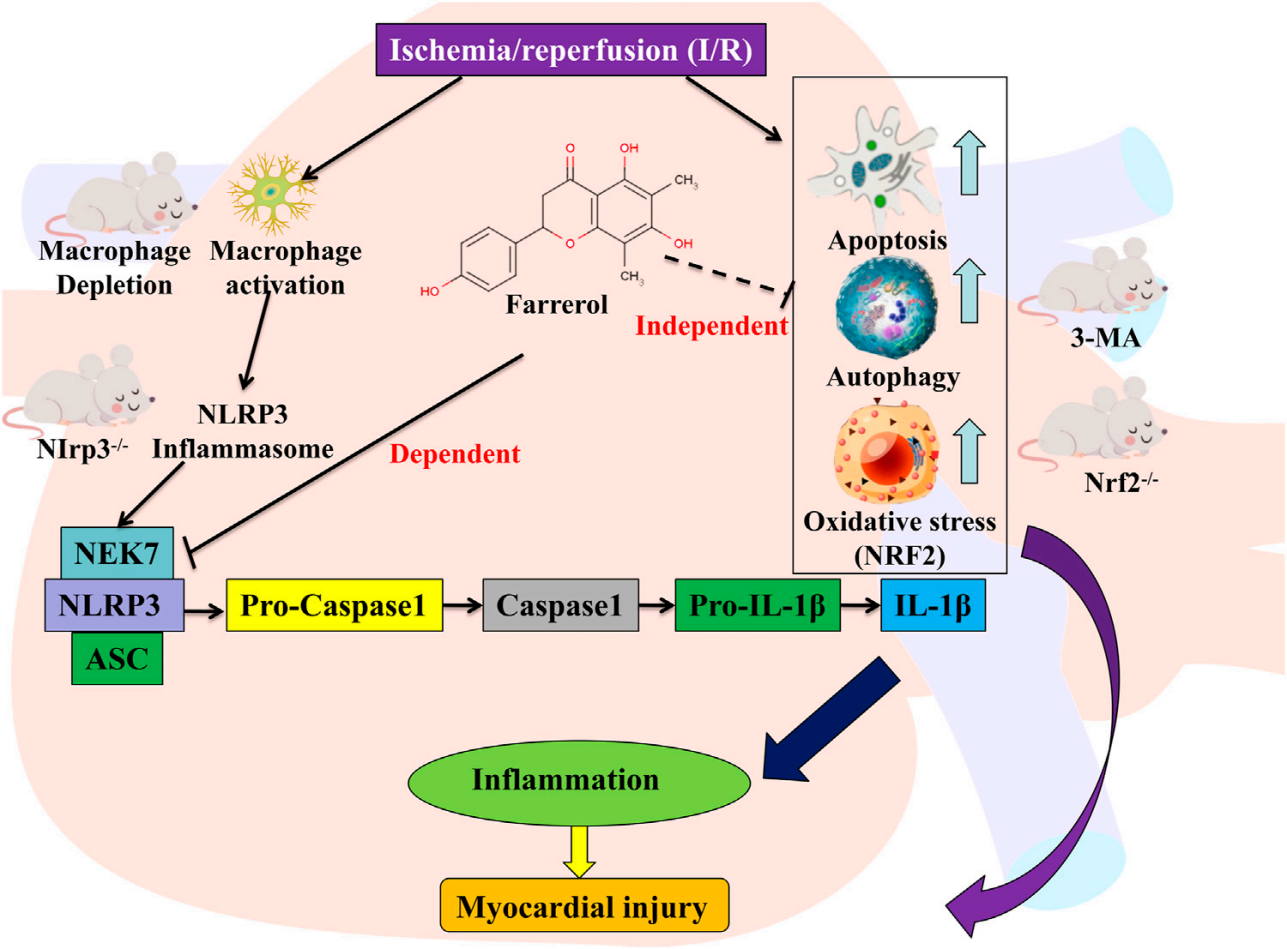
Graphic abstract for major findings and proposed mechanism. FA inhibits the NEK7-NLRP3 interaction, thereby inhibiting NLRP3 inflammasome assembly and activation, resulting in an effective inhibition to alleviate myocardial ischemia/reperfusion injury. Schematic representation of the mechanism of action of FA.

In summary, the experimental results of this study showed that the protective effect of FA did not through Nrf2-dependent or autophagy-dependent pathways, but may be due to its effect on macrophages rather than a direct effect on cardiomyocytes. After the depletion of macrophages, the protective effect of FA decreased dramatically suggesting that FA indirectly protects cardiomyocytes by targeting macrophages. Further studies showed that the protective effect of FA against I/R-induced myocardium injury involves blocking NLRP3 inflammasome assembly by interfering with the interaction between NLRP3 and NEK7, thereby inhibiting NLRP3 inflammasome activation (Figure 11). In conclusion, this study demonstrates a novel role of FA as a potential myocardial protective agent in the protection and treatment of I/R-induced myocardial injury by targeting NLRP3 in macrophages.

## Data Availability

The original contributions presented in the study are included in the article/Supplementary Material, further inquiries can be directed to the corresponding author.
